# Polypy: A Framework to Interpret Polymer Properties from Mass Spectrometry Data

**DOI:** 10.3390/polym16131771

**Published:** 2024-06-22

**Authors:** Vitor Vlnieska, Ankita Khanda, Evgeniia Gilshtein, Jorge Luis Beltrán, Jakob Heier, Danays Kunka

**Affiliations:** 1 Laboratory for Functional Polymers, Swiss Federal Laboratories for Materials Science and Technology (EMPA), Überlandstrasse 129, 8600 Dübendorf, Switzerland; 2 Laboratory for Thin Films and Photovoltaics, Swiss Federal Laboratories for Materials Science and Technology (EMPA), Überlandstrasse 129, 8600 Dübendorf, Switzerland; 3 Integrated Quantum Optics, Institute for Photonic Quantum Systems (PhoQS), Paderborn University, Warburger Str. 100, 33098 Paderborn, Germany; 4 Department of Electrical and Photonics Engineering, Technical University of Denmark, Anker Engelunds Vej 101, 2800 Kongens Lyngby, Denmark; 5 Institute of Microstructure Technology, Karlsruhe Institute of Technology (KIT), Hermann-von-Helmholtz-Platz 1, 76344 Eggenstein-Leopoldshafen, Germany

**Keywords:** polymers, python, polymer characterization, polymer chain distribution, mass spectrometry, gel permeation chromatography, aryl resin, mass polymerization

## Abstract

Mass spectrometry (MS) is a robust technique for polymer characterization, and it can provide the chemical fingerprint of a complete sample regarding polymer distribution chains. Nevertheless, polymer chemical properties such as polydispersity (Pd), average molecular mass ( $M_{n}$ ), weight average molecular mass ( $M_{w}$ ) and others are not determined by MS, as they are commonly characterized by gel permeation chromatography (GPC). In order to calculate polymer properties from MS, a Python script was developed to interpret polymer properties from spectrometric raw data. Polypy script can be considered a peak detection and area distribution method, and represents the result of combining the MS raw data filtered using Root Mean Square (RMS) calculation with molecular classification based on theoretical molar masses. Polypy filters out areas corresponding to repetitive units. This approach facilitates the identification of the polymer chains and calculates their properties. The script also integrates visualization graphic tools for data analysis. In this work, aryl resin (poly(2,2-bis(4-oxy-(2-(methyloxirane)phenyl)propan) was the study case polymer molecule, and is composed of oligomer chains distributed mainly in the range of dimers to tetramers, in some cases presenting traces of pentamers and hexamers in the distribution profile of the oligomeric chains. Epoxy resin has $M_{n}$ = 607 Da, $M_{w}$ = 631 Da, and polydispersity (Pd) of 1.015 (data given by GPC). With Polypy script, calculations resulted in $M_{n}$ = 584.42 Da, $M_{w}$ = 649.29 Da, and Pd = 1.11, which are consistent results if compared with GPC characterization. Additional information, such as the percentage of oligomer distribution, was also calculated and for this polymer matrix it was not possible to retrieve it from the GPC method. Polypy is an approach to characterizing major polymer chemical properties using only MS raw spectra, and it can be utilized with any MS raw data for any polymer matrix.

## 1. Introduction

Polymers’ physico-chemical properties are directly correlated with the mass distribution profile, the degree of functionalization, and the shape of the chain distribution. The characterization of polymers is highly dependent on the polymerization synthesis technique and the polymer chemical structure itself, which makes the characterization protocol quite complex [1,2].

In the last four decades, significant advancements made in devices based on mass spectrometry (MS) have resulted in fast and accurate protocols for macromolecule characterization, mainly due to the maturing of soft ionization procedures based on electrospray ionization (ESI), laser desorption ionization (LDI), and matrix-assisted laser desorption ionization (MALDI). For most polymer samples, it is now possible to obtain broad MS spectra and avoid fragmentation of the molecules during the ionization process [1,2,3,4]. Since the advent of these techniques, one can observe that classic characterization techniques such as size exclusion chromatography (SEC) and liquid adsorption chromatography (LAC) are often no longer sufficient to access the required information for polymer characterization [3].

**Figure 1:**
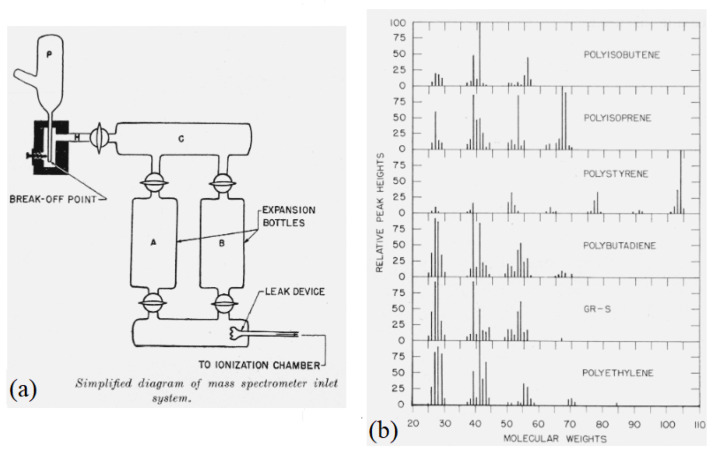
State of the art of mass spectrometry in the 1950s. (**a**) Single inlet sample injection for mass spectrometry ionization [13]. (**b**) Mass spectra of diverse polymers [14].

Between the 1940s and 1960s, the main application of emission, fluorescent, and photometric mass spectrometric methods was in the detection of chemical sample impurities [5]. However, at that time these methods were already being investigated for polymer characterization. Anufriev et al. (1965) applied MS as a characterization method to investigate the thermal degradation mechanism of polymethylmethacrylate (PMMA) [6], and they built up their findings based on a similar approach for the thermal characterization of polystyrene [7] and polyethylene [8]. Charlesbby and Callaghan (1958) mentioned the advent of mass spectrometry as an auxiliary method for analyzing side chains of polyethylene [9]. It is also interesting to note the “Bibliography on Mass Spectrometry”, in which most of the MS applications in “Section D—Applications to Organic Chemistry” were oriented to the petroleum industry [10]. Additionally, in the 1940s and 1950s, several reports present MS applications for polymer characterization; for example, Madorsky and Straus (1948) [11] describe a characterization method using mass spectrometry for polystyrene pyrolysis fractions, Ciapetta et al. (1948) utilize mass spectrometry to evaluate fractions of butylene polymers [12], and Wall (1948) presents a mass spectrometric investigation of the thermal decomposition of several vinyl and diene polymers [13]. Figure 1 depicts a typical inlet apparatus and MS spectra from diverse polymer samples.

In the 1970s, noticeable developments in MS instrumentation and methods led to a vast acceptance of MS in almost every field of research, including pharmaceuticals, food and flavoring, ecology, pharmacology, clinical medicine, polymer science, and many others. MS methods, in combination with gas chromatography (hyphenated GC-MS), became an indispensable tool for biochemical analysis, as well as chemical ionization and mass fragmentography methods, the main MS protocols for which emerged in the 1970s. At the end of this decade, it was clear to the research community that there were challenges related to the MS ionization process due to the ion formation step. As there are diverse ways of providing energy to a certain molecule to generate its ion species, naturally, the mass spectrum of it may be uniquely bound to the ionization conditions and/or ionization technique. That said, a molecule usually presents a completely different MS spectrum if the ionization protocol is changed. In addition to this challenge, there are multiple ionization pathways, which can be classified into two kinds: molecular ionization and fragment ionization. Molecular ions are chemical species missing electrons at the valence electron shell, which means that no chemical bond cleavage happens. In cases where sufficient energy is available during the ionization process, the majority of organic molecules do not lose a second electron at the valence shell; instead, they undergo a fragmentation process, splitting the molecules into several other chemical species and resulting in a fragmentation pattern known as the ‘fingerprint’ of the initial molecule species [10,11,15,16].

The middle of the 1970s was also marked by the coupling of MS with liquid chromatography (LC-MS), combining high MS sensitivity with LC separation efficiency for non-volatile molecules. Definitions of on/off-line were used for different LC-MS combination setups, where off-line stands for MS analysis after LC separation. In this technique, the LC mobile phase is fractioned into different retention times, isolating each desired set of molecules or single molecule. Afterward, each fraction is characterized by MS. The on-line system solves the challenge of inserting low or non-volatile molecules into a high vacuum system to achieve the ionization process [17].

As MS techniques matured, it became clear that mass spectrometry does not deal with a well-defined property of molecules, but depending on the ionization process, distinct results and spectra can be obtained. Based on this knowledge, one has to select the appropriate ionization technique that will be the most suitable for the structure determination of a target molecule. From the end of the 1980s to 2000, new ionization protocols led to the development of the soft ionization method, which aims to avoid the molecule fragmentation process. Solid-state ionization processes, including Laser Desorption Ionization (LDI) and Matrix-Assisted Laser Desorption Ionization (MALDI), and also the liquid state process of electrospray ionization (ESI), are the most well known soft ionization techniques [1,4,18,19,20,21,22].

In the 21st century, developments in software and hardware for the better coupling of several techniques led to innovative approaches using, for example, Ion Mobility Spectrometry coupled with Mass Spectrometry (IMS-MS). Two-dimensional chromatographic setups have also been studied specifically for polymer characterization, providing simultaneous information regarding molar mass and chemical functionality, such as co-polymer composition ratio and polymer chain end-groups.This has been achieved by taking advantage of liquid adsorption chromatography at critical conditions (LACCC), obtaining separation in polymer chain functionalization and combining it with size exclusion chromatography (SEC), which separates molecules by molar weight [1,3,23,24].

**Figure 2:**
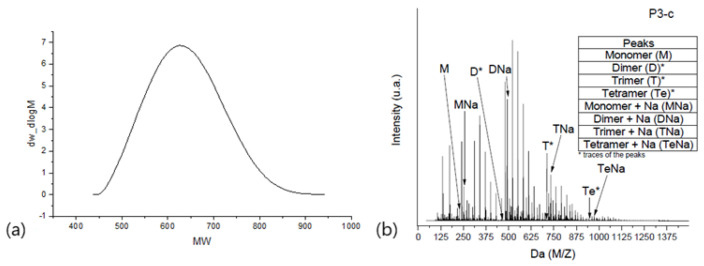
Chromatogram and spectra comparison between GPC and ESI-tof-MS characterization for oligomeric aryl resin. (**a**) Aryl resin GPC chromatogram. (**b**) Aryl epoxy ESI-tof-MS spectra.

Although MS-hyphenated techniques have become very powerful for polymer characterization, one has to deal with spectral complexity to elucidate a sample, as depicted in Figure 2b.

In Figure 2a, oligomeric phenolic resin is characterized by means of gel permeation chromatography (GPC), and in Figure 2b, the same aryl resin is characterized using ESI-tof-MS. Notably, for this specific case, GPC usually does not provide enough resolution to separate each repetitive unit from the oligomer distribution. In such cases, the common overlapping of the oligomer derivative is visible only by analyzing the MS spectra. As another example, a simple homopolymer with average molecular mass ( $M_{n}$ ) = 3.3 × 10^3^ g·mol^−1^ and polydispersity (Pd) of 1.27 will present 60 distinct masses, without considering isomer analogs [22,24].

In the last two decades, significant development in custom scripts for MS data interpretation has been achieved. The literature presents several approaches to tackle the complexity of MS data using distinct programming strategies. Nevertheless, one will note that most applications were developed towards biological samples. Hu et al. (2023) presented Mass-Suite script, a Python interpreter designed to analyze high-resolution MS for water quality assessment and general environmental application using machine learning technique for peak assignment [25].

Davila et al. (2022) used machine learning to better interpret chemical fingerprint workflows of the same molecule/target that are characterized from different sample sources [26]. Nikolopoulou et al. (2022) developed TrendProbe script to characterize samples via LC-HRMS through a non-target screening method combined with deep learning and neural network programming strategies, using R programming language [27].

Helmus et al. (2021) reported patRoon, an open-source platform to help in the interpretation of non-target screening MS datasets. The script can be used through R, C++, and JavaScript programming languages [28]. Liebal et al. (2020) presented an overview of machine learning methods applied for metabolomic characterization from MS datasets [29]. Melnikov et al. (2020) describe Peakonly, a script designed based on a deep learning strategy for accurate peak detection in high-volume MS datasets. The script was written in Python [30].

Riquelme et al. (2020) reported TidyMS, a Python package designed for LC-MS data of untargeted metabolomic characterization [31]. Levitsky et al. (2019) reported Pyteomics 4.0, a Python interface for proteomics data characterization [32].

The MS-DIAL script was presented by Tsugawa et al. (2015) for the identification and quantification of small molecules through mass spectral deconvolution, using MS excel sheets [33]. Röst et al. (2016) developed an OpenMS framework based on C++ and Python to overcome issues regarding the volume and complexity of high throughput MS data, for example in the field of proteomics [34].

Improvements to large-scale metabolomics data using R programming were presented by Uppal et al. (2013) using xMSanalyzer script, in which the automated processing of metabolomics is possible [35]. Pluskal et al. (2010) presented MZmine 2, a Java script designed to overcome the processing of complex profiling biological samples, such as proteomics, genomics, and metabolomics [36].

Over the course of the 21st century, MS methods have established themselves as fundamental techniques in polymer science, providing in-depth information about polymer samples. However, they have also raised new challenges in interpreting complex data, making it clear that the interpretation of the dataset has become more complex and time-consuming. In order to help with this challenge, a Python script is proposed to interpret and calculate fundamental polymer properties from MS raw spectra. Our script implements peak detection and area distribution calculations regarding repetitive units, as a result of combining the filtering of MS raw data using Root Mean Square (RMS) calculation with molecular classification based on theoretical molar masses. The script also integrates graphic visualization tools for data analysis.

## 2. Materials and Methods

Aryl resins (poly(2,2-bis(4-oxy-(2-(methyloxirane)phenyl)propan) are oligomer chains distributed mainly in the range of dimers to tetramers, and in some cases presenting traces of pentamers and hexamers in the distribution profile of the oligomeric chains. $M_{n}$ is 607 Da and Pd is 1.015, both given by GPC. Aryl resins were synthesized previously, and detailed information on synthesis and characterization is presented by Vlnieska (2019) [37].

### 2.1. Electrospray Ionization–Time of Flight–Mass Spectrometry (ESI-µTOF-MS)

Samples were prepared in a concentration range of 10^−6^ mol·mL^−1^, and acetone was the solvent. Spectra were acquired using a microTOF-QII spectrometer (Bruker, Karlsruhe, Germany). Acquisition was set to positive mode, 5.5 × 10^3^ V, with a nebulizer with no pressure, dry gas flow at 3.0 mL·mL^−1^, a dry temperature of 90 °C, transfer system with radio frequency (RF) 1 and RF 2 at 200 VPP, Hexapole at 100 VPP, ion energy of 3.0 eV, collision energy at 12.0, collision RF of 250 VPP, transfer time of 70 µs, and pre-storage of 5.0 µs. The mass range was initially recorded from 10^2^ to 10^4^ M·Z^−1^. After not observing any peaks in the high-mass region, spectra were then recorded in a range from 10^2^ to 2.5 × 10^3^ M·Z^−1^.

### 2.2. Spectra Interpretation through Python Algorithm—Polypy

Spectrometric raw data were imported from text files and processed in Python. First, the background signal was removed from datasets. All values below the defined threshold were excluded from further processing. In this study, the threshold was set to the root mean square error (RMSE). Mass peaks for each oligomer (neat units) and its derivatives and adducts were found by comparing detected values with theoretically derived masses, based on each product’s chemical composition. Additionally, possible mass variation due to isomers was included as tolerance for mass values for products from monomers to tetramers, leading to most peaks being able to be detected by the algorithm. The Polypy interpreter main logic can be represented by the following steps:

(I) Theoretical masses calculation;
(II) MS raw data reading and filtering;
(III) Background signal noise filtering;
(IV) Classification of neat, derivative, and side-product peaks;
(V) Separation of repetitive unit regions and distribution percentage calculation;
(VI) Polymer properties calculation;
(VII) Polypy and MS caveats.

## 3. Results and Discussion

### 3.1. Aryl Resins—Neat, Derivative, and Adduct Product Definitions

**Figure 3:**
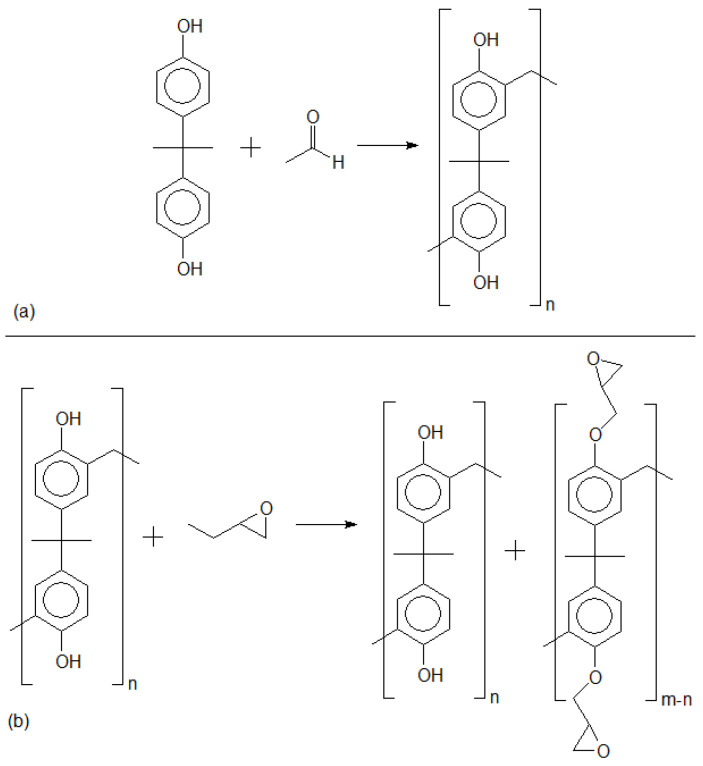
Two-step synthesis of poly(2,2-bis(4-hydroxyphenyl)propan). (**a**) Electrophilic aromatic substitution, applying formaldehyde as an electrophile. (**b**) Aryl resin epoxydation (alkylation), applying 1-Chlor-2,3-epoxypropan as alkylant agent.

The mass polymerization (bulk polymerization) reaction technique provides low control of the polymer chain nucleation and propagation [38]. Nonetheless, for a diverse set of molecules, including the molecule in this study, (2,2-bis(4-hydroxyphenyl)propan)), mass polymerization is an effective technique to synthesize polymer chains. In this regard, one has to accept the formation of derivative compounds during polymerization reactions. Figure 3 presents the mass polymerization reaction from 2,2-bis(4-hydroxyphenyl)propan.

**Figure 4:**
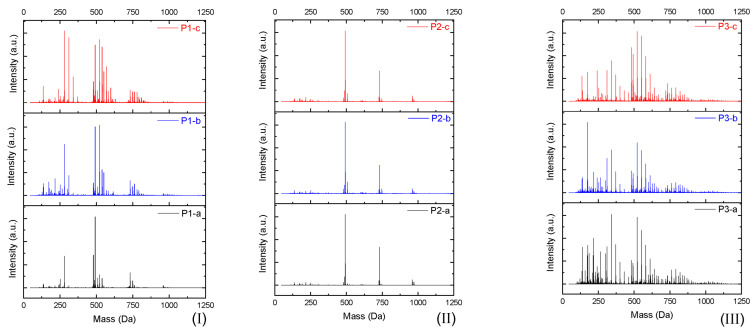
The bis(4-oxy-(2-(methyloxirane)phenyl)propan MS spectra in different synthesis parameters. Reproduced with permission from Vlnieska (2019) [37]. (**I**), (**II**) and (**III**) represent the same reaction system (triplicates), applying different values for molar ratio between alkylation agent:monomer, temperature, time, and solvent volume.

Mass polymerization through the electrophilic aromatic substitution (EAS) of 2,2-bis(4-hydroxyphenyl) propane can generate distinct profiles of oligomers. Depending on synthesis parameters, different profile distributions of the polymer/oligomer chains will be obtained. Figure 4 presents three MS spectra of poly(2,2-bis(4-hydroxyphenyl)propan), each of them a triplicate reaction, and one can observe different oligomer profiles. Although using the same synthesis protocol, due to the different molar ratio between reagents, temperature, reaction time, and catalyst amount, distinct results are obtained [37].

MS spectra provide a detailed chemical fingerprint of the polymer chains, presenting every single difference in molecular weight (Da) within the polymer distribution. Together with a theoretical mass assignment of the oligomer formation, one can define the following chemical structure categories:

- Repetitive units, considered neat products from polymerization.
- Derivatives, which are repetitive units added by an n numbers of hydroxymethyl groups.
- Adducts, which are usually one of the categories above, with sodium atoms added at phenolic positions.

**Figure 5:**
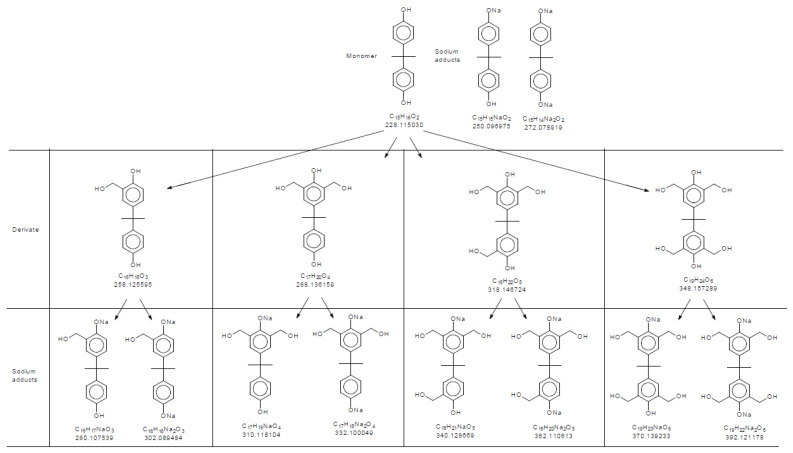
The 2,2-bis(4-hydroxyphenyl)propan mass polymerization derivative and sodium adduct representation based on monomer molecules.

Sodium adducts are usually products specifically generated during the atomization process of mass spectrometry, and which have to be considered in order to characterize the polymer system [39]. Figure 5 presents derivatives and adducts from the monomer molecule. Figures S1 and S2 in the Supplementary Material present dimer and trimer mechanism formations regarding derivative and adduct products.

**Figure 6:**
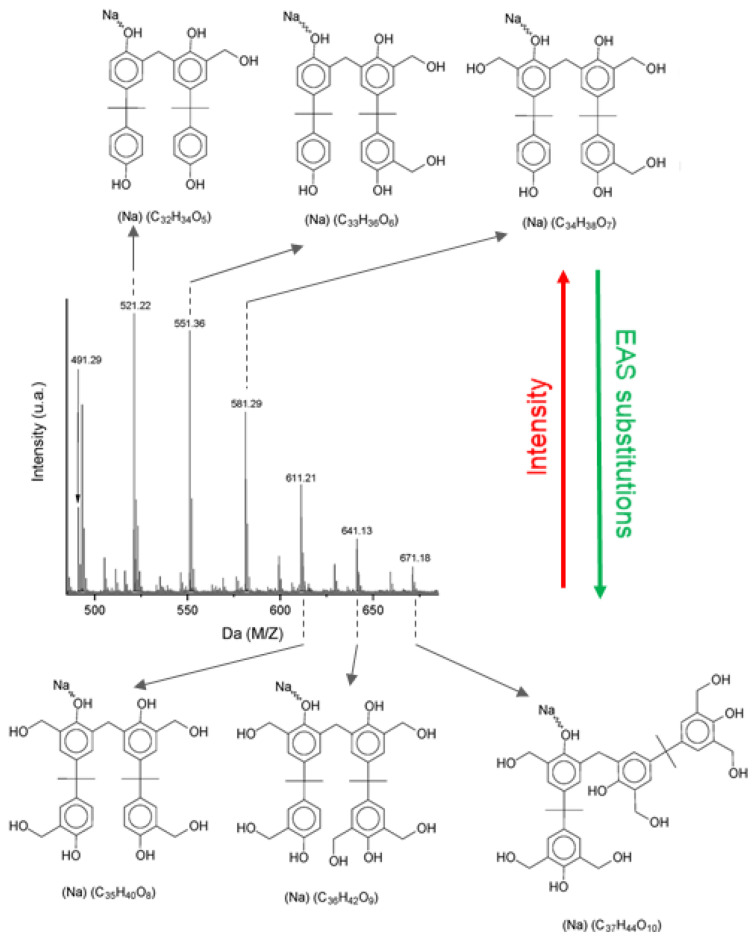
MS spectra at dimer region from sample P3-C and derivative molecular structures of the mono sodium adduct dimer. Increase in EAS substitutions results in lower intensities in MS spectra.

It is worth noting for this molecule that, due to the higher energy activation in *meta* positions, only *orto* positions are considered for reaction in aromatic rings, as depicted in Figure 3a. That said, each monomer molecule will have four positions to be alkylated through EAS mechanism. [37]. Although *meta* positions are not considered for polymerization, MS data are still expected to be considerably dense. Previously evaluated experimental data have shown that the more chemical substitutions take place, the lower the intensity of sodium adducts and hydroxymethyl derivatives. This result indicates that either the atomization process is less effective for highly substituted repetitive units (known as the suppression effect of soft MS ionization setups), or the amount of those products is lower than that produced during synthesis, following EAS kinetics, which is the more probable hypothesis. Figure 6 presents evidence regarding the amount of hydroxymethyl group substitutions versus spectra intensity.

One can see that the suppression effect for this polymer matrix is not significant due to some pieces of evidence: (1) The polymer matrix is composed only of oligomers (range of 230–1500 Da), which shall not promote a significant suppression effect. (2) There is a linear relationship of the intensities x aryl ring substitutions at the spectra in Figure 6 [40]. (3) GPC data support this statement. If the suppression effect is strong enough in higher molecular weights, $M_{n}$ would be of higher value in GPC analysis, which is not the case for this polymer matrix.

**Figure 7:**
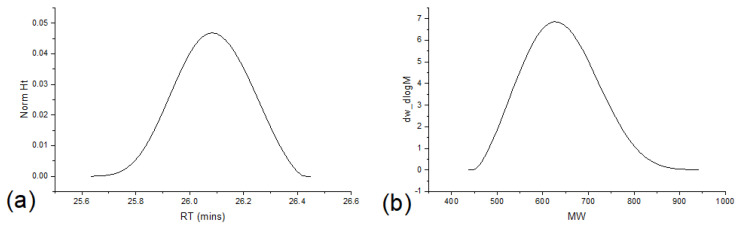
Aryl resin chromatogram. (**a**) Single peak in retention time dimension. (**b**) Oligomeric chain mass distribution.

The interpreting and correlating of peak masses vs. chemical structure demands a considerable amount of time, and besides, one has to know the reaction mechanism of the analyzed material. After such an effort, it could be worthwhile to extract additional information from the MS spectra. In the case of polymer characterization, chain size measures and polymer distribution, such as polydispersity, can be calculated from MS data, and scripts designed for this purpose can be an efficient approach to retrieve polymer properties from MS raw data. Polymer properties are usually characterized via GPC, and as a standard comparison, Figure 7 presents the P3-b sample chromatogram and Table 1 presents its calculated oligomer properties.

For oligomer characterization, it is common to observe results as seen in Figure 7, where characterization is resumed in one single peak. Low resolution is usually found, and attributed to the column properties and GPC settings and optimization. Typically, GPC systems are designed for high molar mass characterization, and GPC setting optimization will most likely not be effective to resolve this chromatogram into each repetitive unit. Thus, small oligomer chains are poorly characterized by means of GPC.

### 3.2. Polypy Interpreter

(I) *MS theoretical masses calculation*

**Figure 8:**
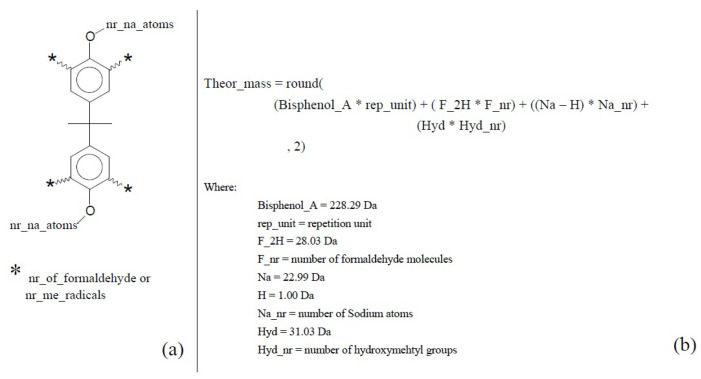
Aryl resin theoretical mass calculation. (**a**) Chemical structure and expected substitution positions. (**b**) Simplified theoretical mass equation extracted from polypy interpreter.

Theoretical masses for aryl resins were calculated based on the aforementioned assumptions, where *meta* positions of the aryl rings are not functionalized and only one methylation happens per repetitive unit. Hydroxymethyl groups can be added at the remaining aryl *orto* positions. Figure 8 depicts how the theoretical masses were calculated based on the monomer structure.

**Figure 9:**
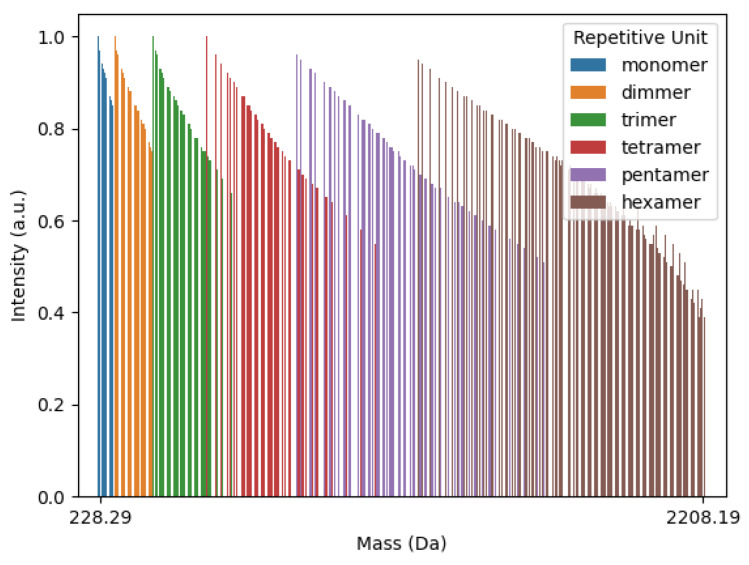
Calculated theoretical masses represented in an MS-like spectrum (plotted using polyps interpreter).

For this study, theoretical masses were calculated from monomers to hexamers, and each single mass value was classified into neat, derivative, and adduct products. Table S1 (Supplementary Material) presents the complete set of mass possibilities generated from the polypy interpreter, whereas in Figure 9 one can observe the entire set of calculated theoretical masses, plotted as an MS-like spectrum.

(II) *Raw data reading*

**Figure 10:**
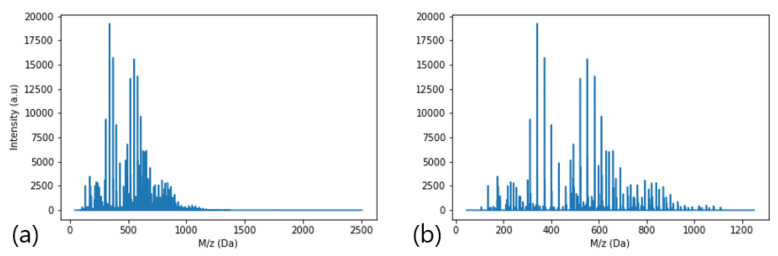
Aryl resin MS spectra. (**a**) Spectrum from MS raw data (**b**) MS spectrum after RMS filtering.

MS data from aryl resin were acquired as described in the Methods section, and afterwards data were evaluated using the polypy interpreter. Spectrometric raw data were imported from text files and processed in Python version 3.9 (main packages: json, math, pandas, matplotlib, numpy, datetime, os, pathlib, pickle, csv, re, matplotlib, seaborn). The graph range of mass values was adjusted based on the intensity values, where the maximum M/Z (Da) value was set after not observing significant intensity, as Figure 10 exemplifies.

(III) *Background signal noise filtering*

In this study, the threshold was set to the root mean square error (RMSE) of the raw data and all values below the defined threshold were excluded from further processing, which, in our case, meant that the RMSE calculation resulted in 16.26 (a.u.). Figure 10a depicts the raw data, and in Figure 10b one can observe results from the RMSE filtering calculation. Also, in Figure 10b, the x-axis was set from 0 to 1250, where peaks were observed. Although RMSE filtering is an efficient approach to avoid background noise signals being calculated within the datasets, for this polymer molecule model, regions of low intensity such as tetramers and pentamers were reduced significantly; therefore, in order to decide which statistical method might be applied for background signal noise filtering, ideally one should know beforehand the major polymer chain distribution properties of the sample.

(IV) *Separation of neat, derivative, and adduct peaks*

**Figure 11:**
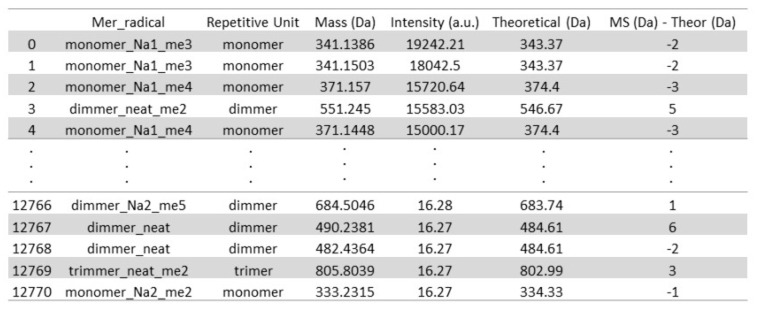
Aryl resin MS spectra classified regarding neat, derivative, and adduct molecules. The table was sorted by the Intensity (a.u.) column.

Neat, derivative, and adduct peaks were distinguished based on the predicted theoretical masses calculated using polypy, as presented in Table S1 (Supplementary Material). The classification of the peaks from experimental data was also carried out with a polypy interpreter. Samples were compared and classified into two main classes: (1) repetitive unit (column: “Repetitive Unit”), and within it (2) neat, derivative, and adduct peaks (column: “Mer radical”), where “NaX” stands for the number of sodium adducts and “meX” for the number of hydroxymethyl groups. In Figure 11, one can observe a snapshot of the table’s head and tail from aryl resins’ experimental MS data after classification using polypy. The script also tracks compared values from experimental and theoretical data (column “Ms (Da)—Theor (Da)”), where one can determine if the given classification is reasonable or not.

(V) *Separation of repetitive unit regions and distribution percentage calculation*

After experimental MS data have been classified, one can then evaluate a diverse set of polymer properties. As mentioned, for this polymer matrix it might be critical to characterize the oligomer chains within its composition. Oligomer regions were classified using the previously calculated theoretical dataset; afterward, the averaged intensity was computed for each of the repetitive unit regions, and a percentage of each region was compared with the total intensity sum of the computation.

**Figure 12:**
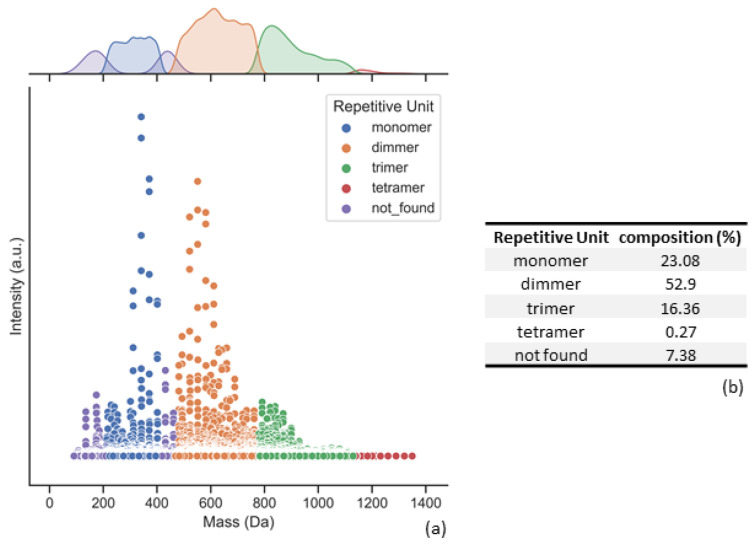
Aryl resin experimental MS data after classification by polypy. (**a**) Spectra represented by repetitive unit regions. (**b**) Percentage distribution of each repetitive unit.

For example, Figure 12 presents oligomer distributions for one of the sample’s sets.

(VI) *Polymer property calculations*

Polymer properties were calculated by applying the following equations to polypy script:

$$\mathrm{Mn}=\frac{\Sigma N_{i}M_{i}}{\Sigma N_{i}}$$

$$\mathrm{Mw}=\frac{\Sigma N_{i}M_{i}^{2}}{\Sigma N_{i}M_{i}}$$

$$M=\frac{\Sigma N_{i}M^{n+1}}{\Sigma N_{i}M_{i}^{n}}$$

$$\mathrm{Mv}={\left[\frac{\Sigma M_{i}^{1+a}N_{i}}{\Sigma M_{i}N_{i}}\right]}^{1/a}$$

where:

$N_{i}$ = number of molecules;

$M_{i}$ = weight of the polymer;

*n = 1 gives M = Mw;*

*n = 2 gives M = Mz;*

*n = 3 gives M = Mz + 1;*

a = Mark–Houwink–Sakurada constant.

**Figure 13:**
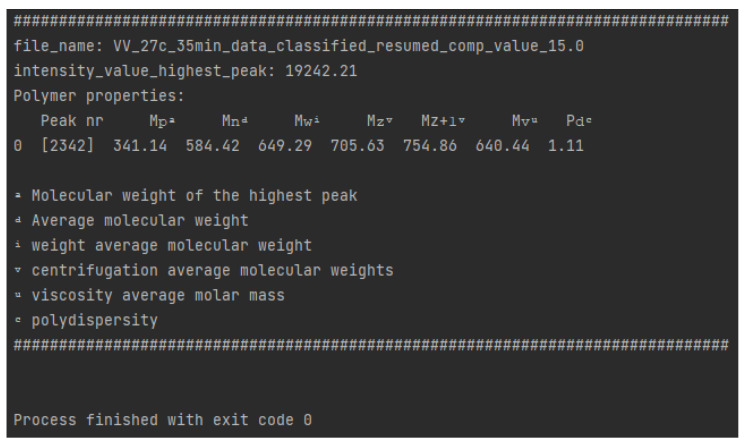
Experimental MS oligomer properties (calculated with polypy).

As $N_{i}$ is usually an internal and relative correlation between distribution fractions of the polymer matrix [41], one can apply the same approach to the script; nevertheless, each mass peak is now identified with a specific structure, leading to an accurate computation of $N_{i}$ × $M_{i}$ , where the precise contribution of each molecule to the average weight of the polymer matrix can be retrieved. For the internal estimation, $N_{i}$ = 10,000 was applied to the most intense peak. Figure 13 presents the calculated polymer properties for the aryl resin using polypy script.

(VII) *Polypy and Mass Spectrometry caveats*

**Figure 14:**
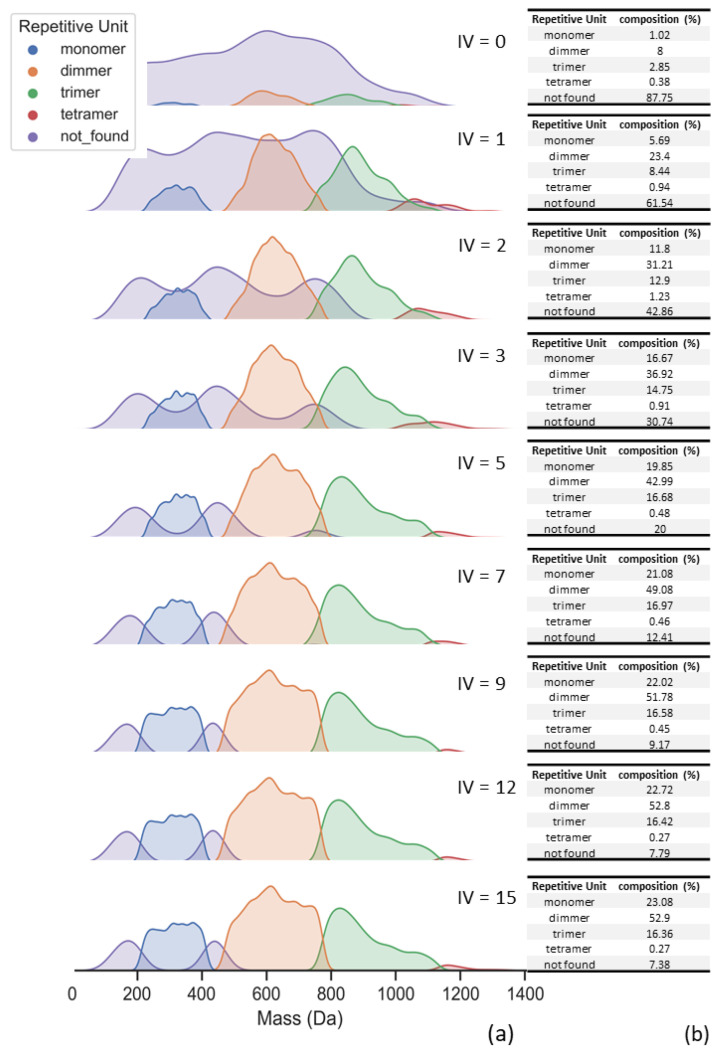
Oligomer distribution of aryl resin. (**a**) Distributions are directly affected by the ionization value limit (IV). (**b**) Percentage calculation of oligomer distribution.

The comparison between theoretical datasets and experimental data requires a limit value to assign each MS peak to a respective theoretical structure. In our script, this comparison was given by the ionization value term, which mimics the number of protons that a certain structure could release during the ionization process in the MS experiment. Figure 14 presents the influence of ionization value for the experimental MS data.

In Figure 14, one can observe a clear distinction between no ionization limit and ionization values above 3. Since the repetitive units have multiple ionization possibilities (see Figure 5), it is reasonable to set the ionization term with values above 10. For example, the complete alkylated aryl monomer would have 6 possibilities of ionization, and the complete alkylated aryl dimer structure would present 10 possibilities of ionization (in these examples, “complete” means alkylation only at *orto* positions of the aromatic rings).

Revisiting the introduction, one has to pay attention to the nature of the MS ionization mechanism. How the ionization energy is transferred to the chemical system is not yet completely elucidated. This results in nonlinear energy transfer to the polymer matrix, and eventually, one range of molecules can become more ionized than others, resulting in nonlinear intensity. This conflicts with the internal estimation method, where $N_{i}$ is defined as the number of molecules for each characterized peak, retrieved using peak intensity as an internal linear comparison. To emphasize good characterization practice, one could obtain a series of MS spectra from the same sample, averaging the intensities, through which the nonlinear ionization transfer energy might be mitigated, and the characterization protocol would be more reliable.

## 4. Conclusions

Polypy is a framework to characterize major polymer properties through mass spectrometric data. The interpreter can be applied to any polymer system characterized by MS.

Nonetheless, differing from other scripts, using polypy, one can calculate the theoretical dataset of values for the target polymer system and use it as a reference dataset. It is worth noting that additional information can be retrieved from the script interpreter, in which, in our case study, the percentage of the oligomer distribution is presented.

In order to make it similar to GPC method, polymer properties such as $M_{n}$ = 584.42, $M_{w}$ = 649.29, $M_{z}$ = 705.63, $M_{v}$ = 754.6, and Pd = 1.11 were calculated using polypy, and the results were the expected ones for this polymer matrix and are consistent with GPC data. Although GPC and MS characterization methods cannot be directly compared, in this study we assumed GPC as an external reference for our script as a proof-of-concept calculation. The literature presents several options like Polypy, and each script tackles MS data either regarding data volume complexity or a specific need from a certain application, like metabolomics, polymer chemistry, and others. Custom script and open source examples such as xMSanalyzer, Mass-Suite, TrendProbe, patRoon, Peakonly, MZmine 2, and OpenMS, and including Polypy, provide the freedom to calculate and interpret any other physical–chemical properties, and additionally, one can apply specific statistics that may be needed for characterization.

## Figures and Tables

**Table 1:** P3-b sample oligomer properties (calculated using the GPC method).

Peak nr	Mp ^a^	Mn ^d^	Mw ^i^	Mz ^v^	Mz+1 ^v^	Mv ^u^	Pd ^c^
1	625	622	631	641	651	630	1.01447

^a^ Molecular weight of the highest peak. ^d^ Average molecular weight. ^i^ Weight average molecular weight. ^v^ Centrifugation average molecular weights. ^u^ Viscosity average molar mass. ^c^ Polydispersity.

## Data Availability

The Raw MS and generated datasets analyzed during the current study are available in the supplementary material. The Python code, herewith named Polypy, is available from the corresponding author upon reasonable request.

## Supplementary Materials

The following supporting information can be downloaded at: https://www.mdpi.com/article/10.3390/polym16131771/s1, Figure S1: Dimer, derivates and sodium adduct formation; Figure S2: Trimer, derivates and sodium adduct formation; Table S1. Theoretical mass values isolated with intensity.
