# Supplementary material for: Polypy: A Framework to Interpret Polymer Properties from Mass Spectrometry Data
Source: Polymers (Basel). 2024 Jun 22;16(13):1771. doi: 10.3390/polym16131771 (PMC11244493; doi:10.3390/polym16131771)
Supplement: Supplementary file 1 [file polymers-16-01771-s001.zip › Figure S1. Dimer, derivates and sodium adduct formation.pdf]

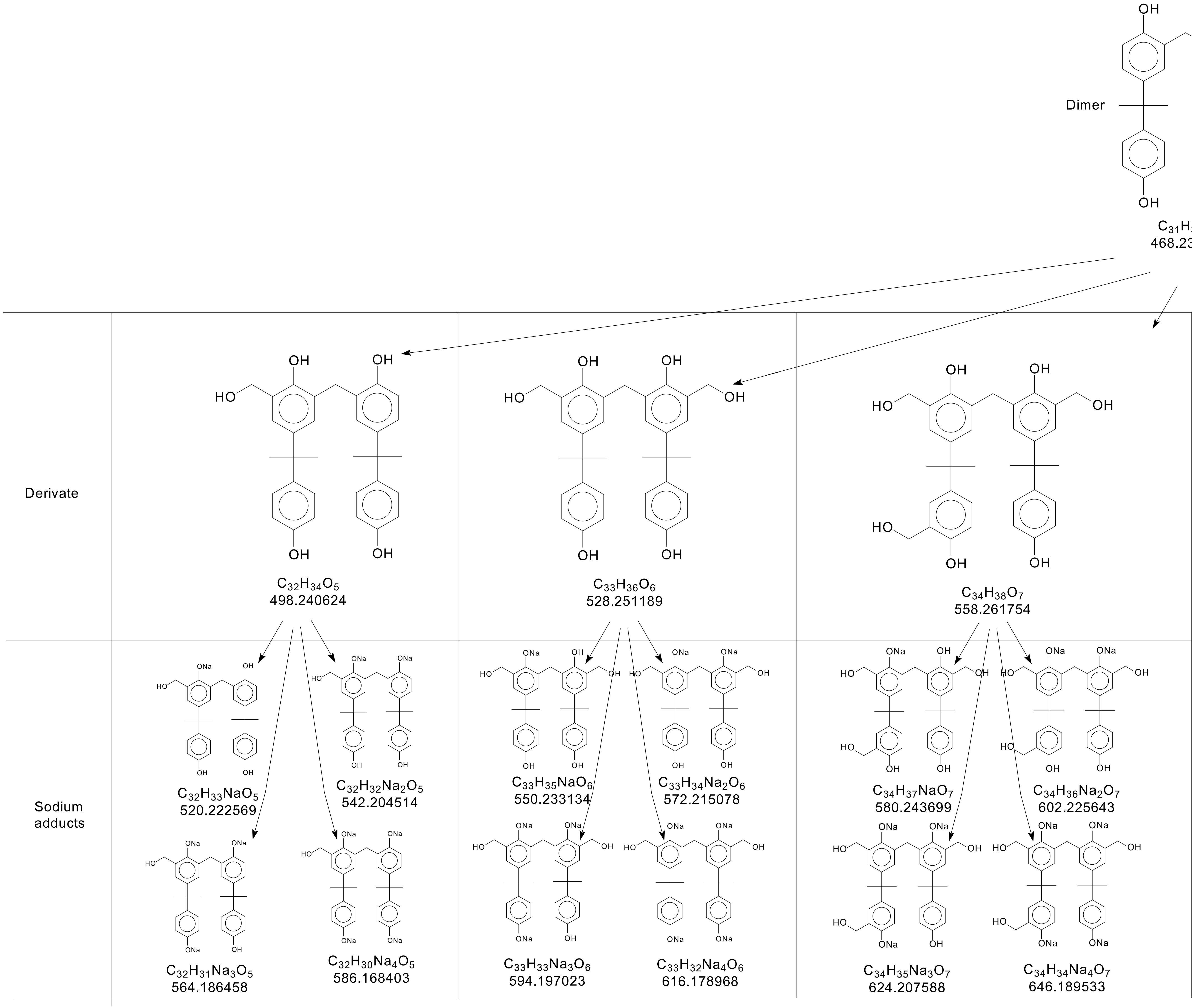
Dimer
Derivate
Sodium adducts
C32H34O5
498.240624
C33H36O6
528.251189
C34H38O7
558.261754
C32H33NaO5
520.222569
C32H32Na2O5
542.204514
C32H31Na3O5
564.186458
C32H30Na4O5
586.168403
C33H35NaO6
550.233134
C33H34Na2O6
572.215078
C33H33Na3O6
594.197023
C33H32Na4O6
616.178968
C34H37NaO7
580.243699
C34H36Na2O7
602.225643
C34H35Na3O7
624.207588
C34H34Na4O7
646.189533

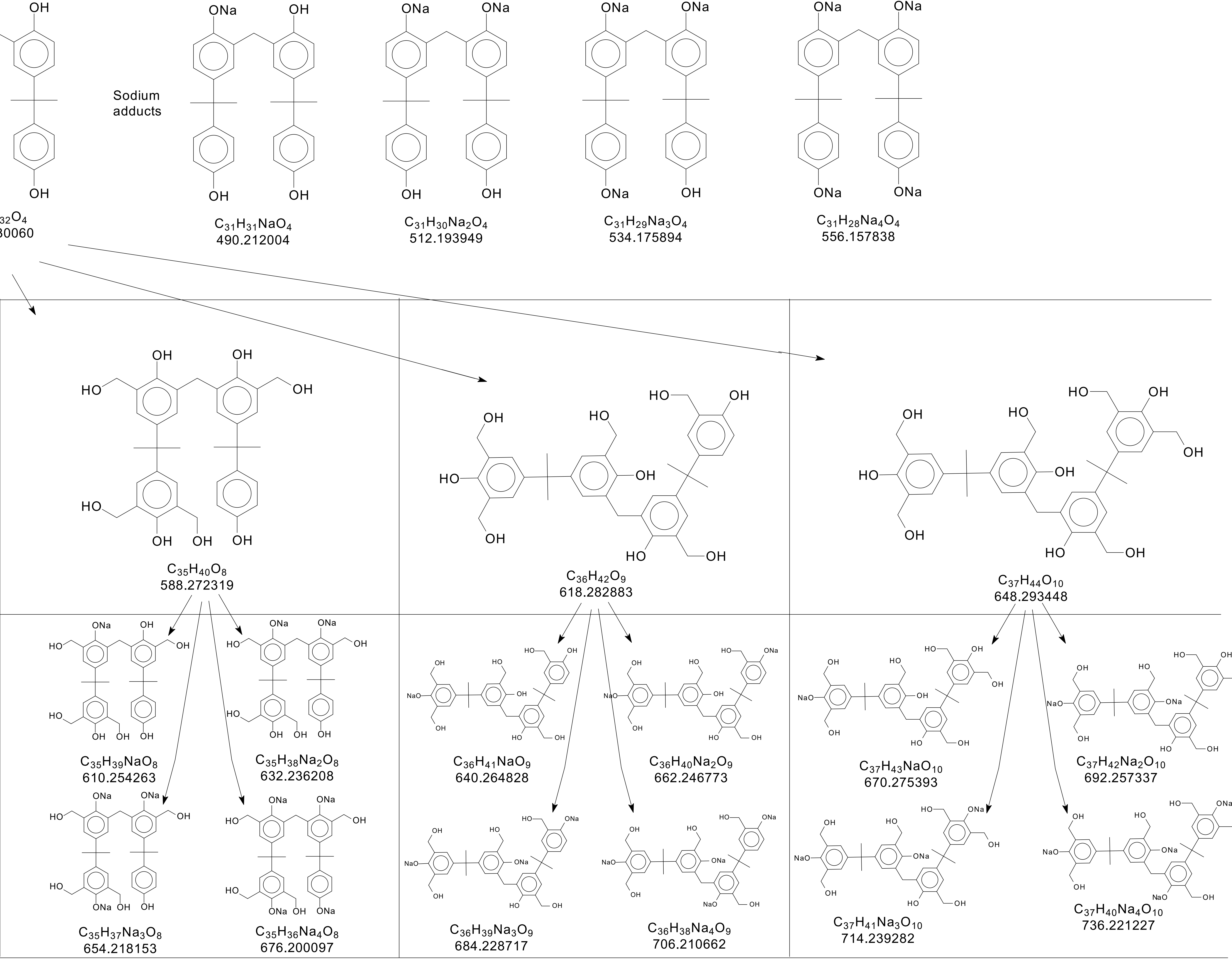
Sodium adducts
$C_{31}H_{31}NaO_4$
490.212004
$C_{31}H_{30}Na_2O_4$
512.193949
$C_{31}H_{29}Na_3O_4$
534.175894
$C_{31}H_{28}Na_4O_4$
556.157838
$C_{35}H_{40}O_8$
588.272319
$C_{36}H_{42}O_9$
618.282883
$C_{37}H_{44}O_{10}$
648.293448
$C_{35}H_{39}NaO_8$
610.254263
$C_{35}H_{38}Na_2O_8$
632.236208
$C_{36}H_{41}NaO_9$
640.264828
$C_{36}H_{40}Na_2O_9$
662.246773
$C_{37}H_{43}NaO_{10}$
670.275393
$C_{37}H_{42}Na_2O_{10}$
692.257337
$C_{35}H_{37}Na_3O_8$
654.218153
$C_{35}H_{36}Na_4O_8$
676.200097
$C_{36}H_{39}Na_3O_9$
684.228717
$C_{36}H_{38}Na_4O_9$
706.210662
$C_{37}H_{41}Na_3O_{10}$
714.239282
$C_{37}H_{40}Na_4O_{10}$
736.221227
