# Supplementary figures and images for: Polypy: A Framework to Interpret Polymer Properties from Mass Spectrometry Data
Source: Polymers (Basel). 2024 Jun 22;16(13):1771. doi: 10.3390/polym16131771 (PMC11244493; doi:10.3390/polym16131771)

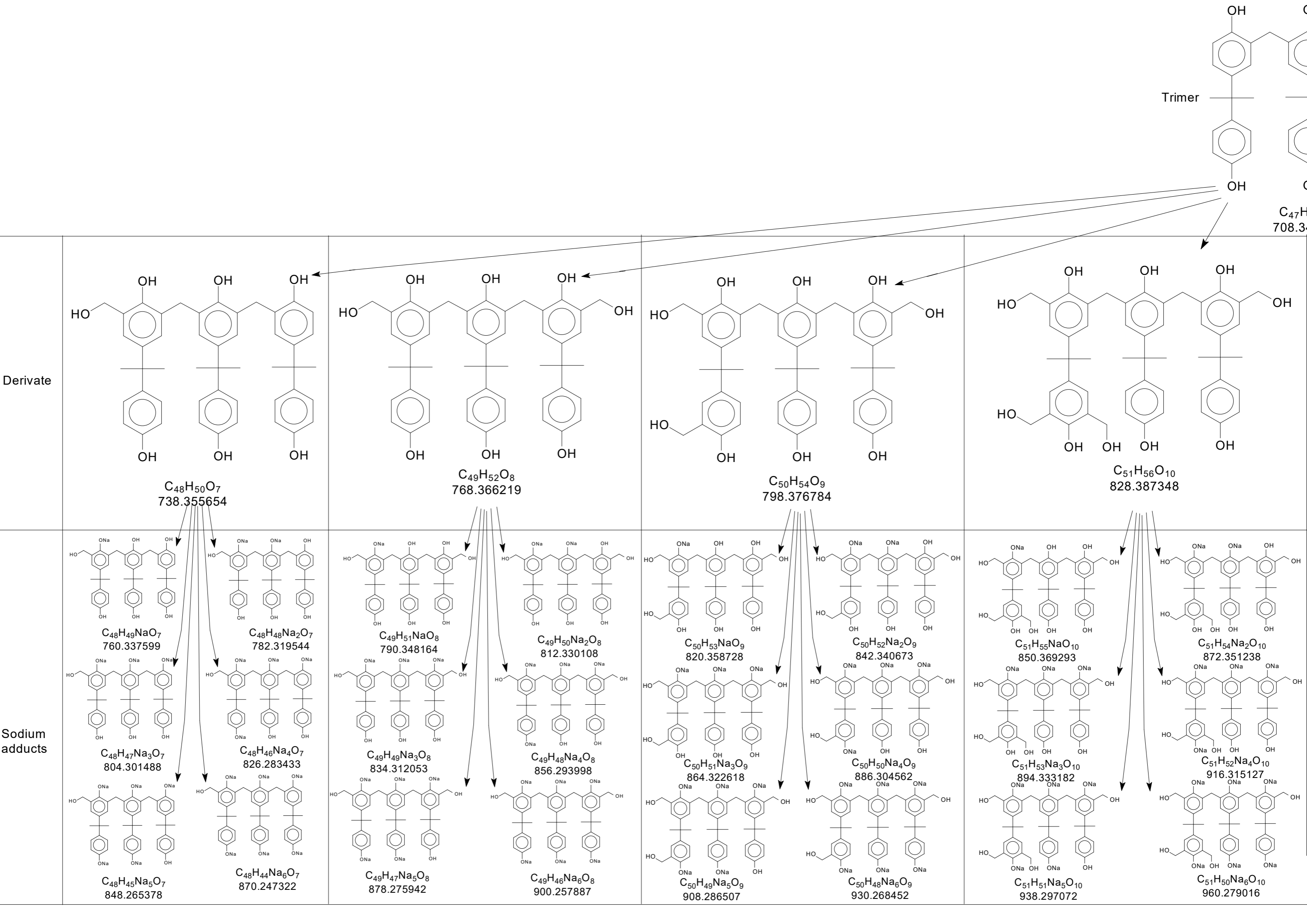

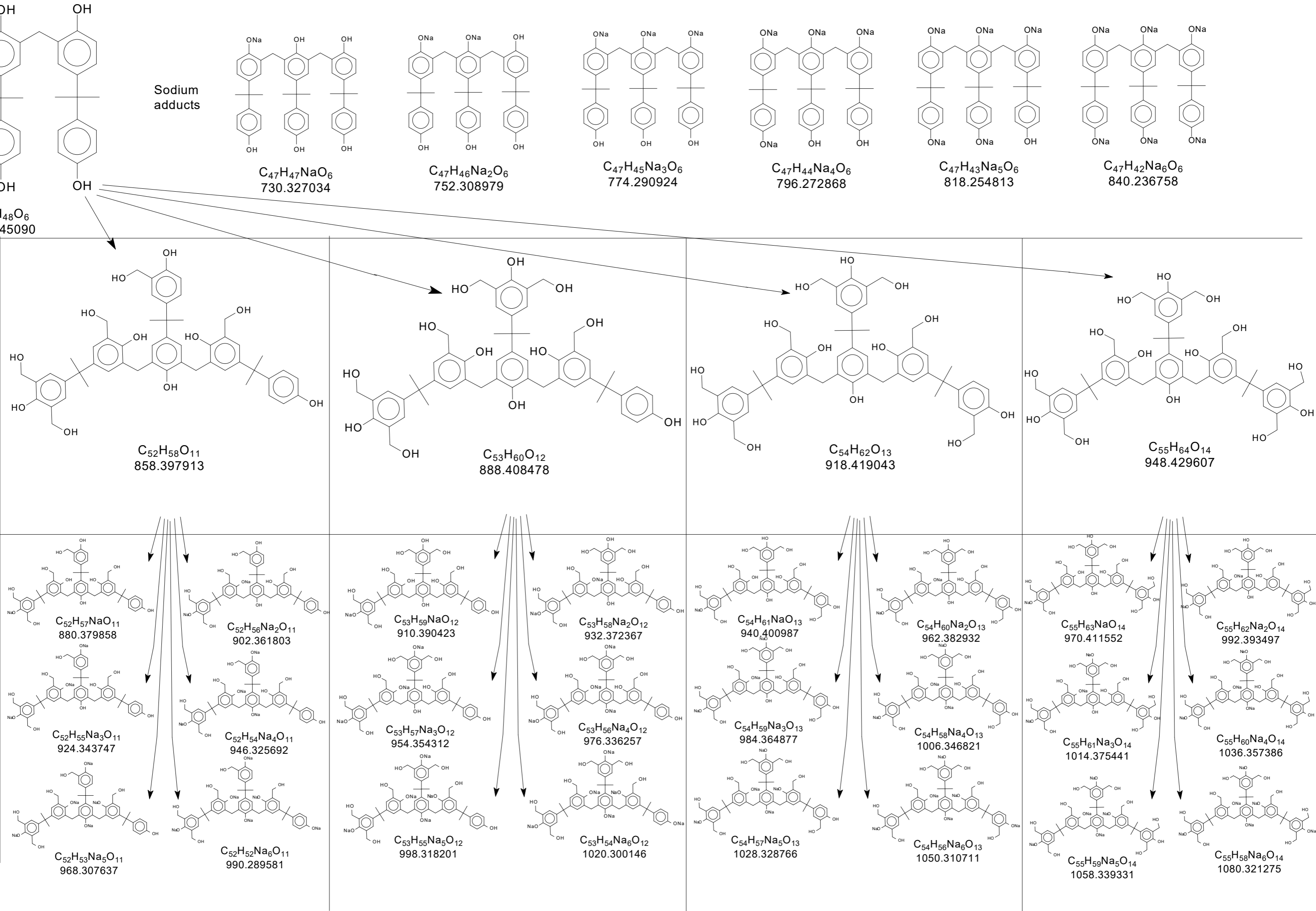

Supplement: Supplementary file 1 [file polymers-16-01771-s001.zip › Figure S2. Trimer, derivates and sodium adduct formation.pdf]
